# Deep Subcutaneous Tick Embedding Following Prolonged Attachment: A Case Report and Mini-Review of Tick Fixation Mechanisms

**DOI:** 10.7759/cureus.102067

**Published:** 2026-01-22

**Authors:** Baogui Wang, Norbert Papdi

**Affiliations:** 1 Family Medicine, Medbase Kloten, Kloten, CHE

**Keywords:** erythema migrans, primary care, subcutaneous embedding, tick bite, tick-borne encephalitis vaccinations (tbe)

## Abstract

Prolonged tick attachment with deep subcutaneous embedding is uncommon in humans but may complicate tick removal in primary care. Firm fixation is mediated by the tick’s barbed hypostome and the secretion of glycine-rich salivary cement, which together anchor the mouthparts within the dermis. Awareness of these mechanisms is important when standard extraction techniques fail. A patient presented with a live, engorged tick deeply embedded in the skin after several days of attachment. The tick’s anterior body and mouthparts were firmly fixed within the dermis, and surrounding erythema migrans was observed. Initial removal using a tick tweezer was incomplete due to persistent fixation, necessitating minor surgical exploration to extract residual fragments. This case highlights that prolonged and unusually firm tick attachment can occur and may require surgical removal when conventional extraction is unsuccessful. Recognizing the mechanical and biochemical mechanisms of tick fixation can guide safe management, prevent retained mouthparts, and support favorable outcomes in primary care.

## Introduction

Tick bites are frequently encountered in primary care, particularly in regions endemic for hard (Ixodid) ticks, such as *Ixodes* species, which serve as vectors for pathogens including *Borrelia burgdorferi* sensu lato and tick‑borne encephalitis virus [1,2]. The risk of pathogen transmission increases with feeding duration, emphasizing the importance of early detection and removal of attached ticks to mitigate disease risk [3,4]. In typical clinical practice, ticks are noticed and removed within hours or a day of attachment. Most attached ticks can be removed using fine‑tipped forceps or tick removal tools; however, incomplete extraction of mouthparts may act as a foreign body, potentially leading to persistent local inflammation, granuloma formation, or secondary infection [5-7]. Understanding the pathophysiological mechanisms that underlie firm tick attachment and strategies for the management of deeply embedded ticks is essential for clinicians in primary care to guide meticulous removal, minimize local complications, and optimize patient outcomes. Prolonged attachment with deep embedding within the dermis is unusual. Here, we report a case in which an *Ixodes* tick remained firmly attached for five days. The case required combined surgical and medical management.

## Case presentation

**Figure 1:**
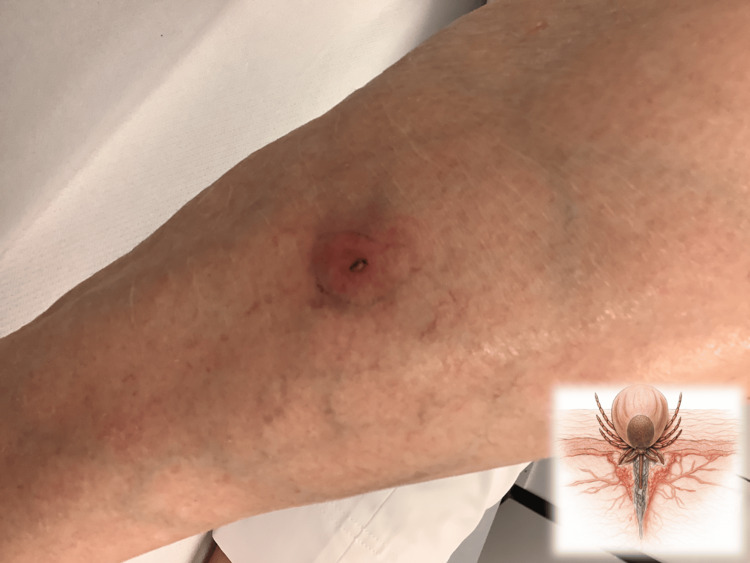
Medical illustration depicting a lateral view of a tick with its body embedded in the dermis. Photographic example with tick in situ obtained on June 16, 2025, of a feeding tick (*Ixodes ricinus*) attached to human skin. The 83-year-old female patient presented to our general practice on June 16, 2025. The image demonstrates the engorged body and the hypostome inserted and anchored within the dermis. The inset in the lower right corner shows a schematic representation generated with the assistance of artificial intelligence, based on a painting by Jian Markus Wang (permission obtained), illustrating deep tick insertion with the hypostome bearing backward-facing barbs and the surrounding glycine-rich cement that stabilizes the tick’s attachment.

An 83-year-old female presented to our general practice on June 16, 2025. The patient performed farming work in the countryside on June 12, which likely represented the point of exposure. Two days later, on June 14, she noticed itching on her lower right leg but did not attempt removal herself, as she felt uncertain. On June 16, she presented to our family medicine clinic. On examination, the patient was afebrile. She exhibited no neurological deficits and reported no significant joint pain, experiencing only mild, intermittent discomfort. A live tick with an engorged abdomen was observed, with over half of its body deeply embedded in the patient’s lower right leg. Clinical examination revealed that the anterior body and mouthparts of the tick were firmly embedded within the dermis. The patient reported mild local numbness without systemic manifestations. Surrounding the site of attachment, a circular erythematous lesion, likely representing an early stage of erythema migrans, was observed (Figure 1).

Investigations

**Figure 2:**
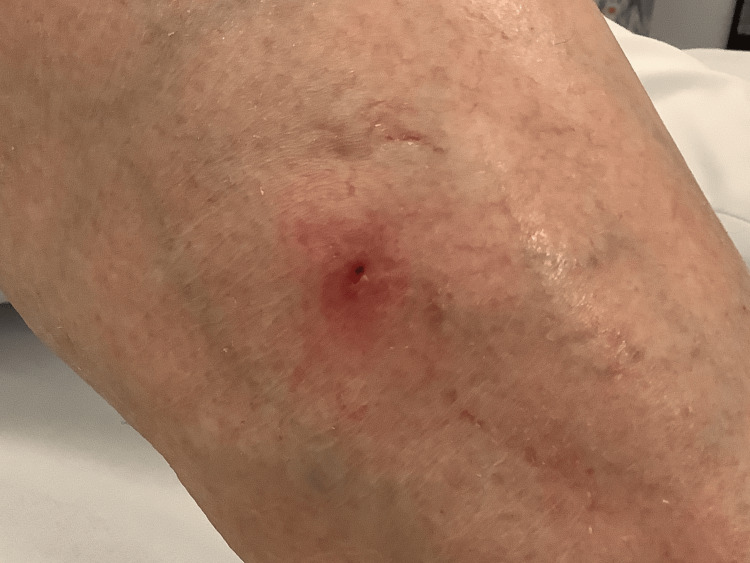
Clinical image after surgical extraction. Photograph taken on June 16, 2025, immediately following tick removal, demonstrating signs of infection as well as erythema migrans.

**Figure 3:**
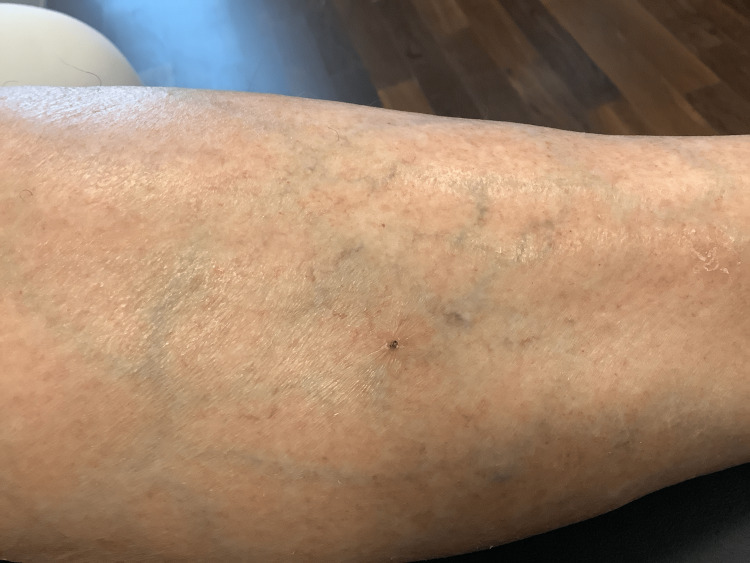
Wound healing. Clinical image demonstrating complete wound closure following surgical extraction and appropriate management, without signs of infection or inflammation.

No laboratory tests were performed at the initial presentation, as clinical examination, together with the presence of erythema migrans, was sufficient for diagnosis. During follow-up, the patient remained afebrile, with pain well controlled and no joint or muscle involvement. Her general condition remained stable, with no neurological symptoms under therapy (Figures 2, 3).

Treatment

Initial removal was attempted using standard tick forceps routinely used in Switzerland (Medbase; stainless steel, steam-sterilizable). Complete extraction proved difficult because part of the tick remained firmly embedded in the dermis. During the attempt, the tick was inadvertently disrupted, leaving a substantial portion embedded in the skin.

The retained fragments were subsequently removed under minor surgical conditions. The affected area was disinfected with Octenisept (octenidine dihydrochloride) and thoroughly irrigated with 0.9% sodium chloride solution. Pain was continuously assessed throughout the procedure and remained adequately controlled without the need for additional analgesic intervention, following prior discussion with the patient. Empiric antibiotic therapy with doxycycline was initiated immediately. The patient also received a tetanus booster and tick-borne encephalitis (TBE) vaccination on the same day. The TBE vaccination is generally recommended in endemic regions.

Management and outcome

The patient was followed clinically on June 17, 2025, June 20, 2025, and June 30, 2025. The wound healed without complications. At the two-week follow-up, the patient was asymptomatic, with complete epithelialization of the site and no signs of local inflammation. There was no evidence of systemic infection, including neurological, musculoskeletal, or joint involvement. Close monitoring confirmed full resolution of the lesion and the absence of secondary complications. The patient, who had no comorbidities, remained free of systemic symptoms, including fever or neurological deficits, throughout the three-month follow-up period.

## Discussion

**Figure 4:**
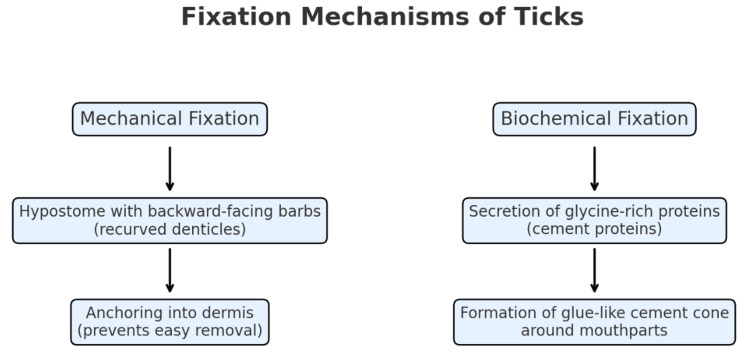
Flow diagram illustrating the mechanical and biochemical fixation of ticks. The diagram illustrates and compares the mechanical and biochemical mechanisms by which ticks anchor to host skin.

This case illustrates an unusually strong attachment of a tick after five days of feeding. While hard ticks can remain attached for several days, they are typically removed earlier, making deep embedding for an extended period uncommon. Reviewing the known mechanisms of tick fixation, i.e., mechanical barbs and biochemical “cement” proteins (Figure 4, Table 1), provides important context for understanding why some ticks resist removal and how to manage such cases safely in family medicine.

**Table 1:** Description of mechanical and biochemical fixation of ticks. The table summarizes the cited references and their principal descriptions corresponding to each mechanical and biochemical mechanism.

Fixation type	Mechanism	References
Mechanical fixation	The hypostome, equipped with backward-facing barbs (recurved denticles), mechanically anchors the tick within the host dermis	Richter et al. (2013) [5]
Biochemical fixation	Secretion of glycine-rich cement proteins that polymerize and harden into a glue-like matrix, reinforcing attachment to the host tissue	Bullard et al. (2016) [8]; Mulenga et al. (2022) [9]; Ganar et al. (2025) [10]

The underlying pathophysiology of the intense fixation observed in our patient could be explained by two complementary mechanisms. First, in mechanical fixation, the tick’s hypostome is lined with recurved (backward-facing) barbs that act like anchors in the skin. These denticles grip the dermis and resist pull-out. As described by TickEncounter, “the tick sinks its hypostome lined with rows of backward pointing barbs, which help hold the tick tightly in the skin while it blood feeds” (web.uri.edu). Scanning electron micrographs by Richter et al. have visualized these hypostomal barbs on *Ixodes* ticks, showing how they embed into the host epidermis [5]. This mechanical “ratchet and barb” mechanism makes simple straight traction removal difficult and can explain why the tick remained stuck in our patient’s skin despite pulling on the body.

Second, in biochemical fixation (cement proteins), in addition to mechanical anchoring, ticks secrete a proteinaceous “cement” from their salivary glands that hardens around the hypostome in the skin. This bioadhesive plug further secures the tick and seals the feeding site. The cement cone is rich in glycine-containing proteins. Bullard et al. reported that cement cones from *Amblyomma americanum* ticks contain numerous proteins, including multiple glycine-rich proteins [6,8]. Mulenga et al. identified and characterized proteins forming the inner core layer of the *Ixodes scapularis* tick attachment cement layer [9]. Wikel [11], Kazimírová and Štibrániová [12], and Sonenshine [13] described how ticks modulate the cutaneous environment to facilitate pathogen establishment. Proteomic studies by Hollmann et al. (2018) found that approximately 19% of cement proteins are glycine-rich, alongside protease inhibitors (12%), mucins, detoxification proteins, and others [14]. Recent reviews emphasize the role of glycine- and hydrophobic-rich adhesive proteins in tick cement. Ganar et al. [10] demonstrated that a glycine-rich tick salivary protein can undergo liquid-liquid phase separation followed by a liquid-to-gel transition, forming a stable adhesive cement cone around the mouthparts. These biochemical “cements” generate a strong, glue-like bond that reinforces the hypostome’s attachment.

Together, the hypostome barbs and cement proteins account for the exceptionally firm attachment observed in this patient. While previous studies [15-17] have described these anchoring mechanisms, clinical reports of prolonged tick fixation in humans remain rare. This dual fixation strategy enables ticks to feed for several days while resisting dislodgement. Such adaptations have evolved to ensure prolonged blood meals despite host defenses (web.uri.edunature.com). Although cases of deep embedding in humans are infrequently documented, they highlight the remarkable biological effectiveness of tick attachment. The study in 2025 by Dr. Takada, titled surgical removal of a tick-bite region without the presence of an insect body, underscores the importance of surgical excision in managing tick bites where the tick body is absent but clinical suspicion remains high [18].

Differentiation between early erythema migrans and a simple bull’s-eye reaction can be challenging in the initial stage. A typical erythema migrans usually develops after at least seven days following tick exposure. In this case, the lesion was defined as erythema migrans and was treated accordingly with first-line antibiotic therapy for Lyme borreliosis. This decision was made in the context of prolonged and deep tick embedding in an 80-year-old patient, where the risk of *Borrelia* transmission was considered increased.

Roupakias et al. reported their institutional experience over a five-year period with surgical tick removal using a minimally invasive technique in which the tick is left in situ during the procedure. This approach offers several advantages. First, the tick is not directly manipulated, which is critical because it minimizes the risk of regurgitation and subsequent transmission of infectious agents into the skin. Second, the technique ensures complete removal of all tick remnants, thereby reducing the likelihood of retained mouthparts. Third, the reported outcomes were excellent: none of the patients treated at their institution, ranging in age from 6 to 65 years, experienced systemic complications or local wound complications following tick removal [19,20].

This method would be difficult to justify in patients presenting within 24 hours of a tick bite. However, in cases of prolonged tick attachment, particularly when the tick is deeply embedded in the skin, the Murtagh Method appears to be a highly interesting and potentially valuable alternative. To date, this technique has not been routinely practiced in our institution. Therefore, we propose conducting a prospective study to evaluate the feasibility, safety, and clinical outcomes of the Murtagh Method in our clinical setting.

## Conclusions

We report an exceptional case encountered in primary care involving deep subcutaneous tick embedding following prolonged attachment. A review of the tick fixation mechanism demonstrates that firm attachment is primarily mediated by the tick’s barbed hypostome and the secretion of glycine-rich salivary cement. These mechanisms represent the most important factors enabling prolonged and deep skin attachment, thereby increasing the risk of pathogen transmission to the human host. In this case, surgical management with complete removal of all retained tick components was essential. The case highlights the importance of recognizing atypical presentations of tick attachment, carefully documenting procedural challenges, and transparently reporting clinical outcomes. Reporting such uncommon presentations contributes to a better clinical understanding of tick attachment mechanisms and their management.
